# Dental politics and subsidy systems for adults in Sweden from 1974 until 2016

**DOI:** 10.1038/bdjopen.2017.7

**Published:** 2017-05-05

**Authors:** Bengt Franzon, Björn Axtelius, Sigvard Åkerman, Björn Klinge

**Affiliations:** 1 Department of Oral Diagnostics, Faculty of Odontology, Malmö University, Malmö, Sweden

## Abstract

**Aims:**

The dental health sector, as part of the Swedish welfare system, originated in 1974. Since then, the dental insurance has undergone three major changes. The aim of this archive study was to study where in the legislative process the dental politics concerning national dental insurance and subsidies were formed.

**Materials and Methods:**

The material, such as Commission of inquiry proposals and Government Bills from four major dental reforms, was collected from the library at the Sveriges Riksdag (Swedish Parliament) and was analysed and structured using a modified version of the Health Field Model.

**Results:**

The views on the fundamental ideas, such as the connection between general and dental health, preventive dentistry, rehabilitation of the mouth and promotion of dental health, were the same over the years. The views on dentistry as a market, when it comes to freedom of prices, have undergone a major change since 1974, but the view on the welfare state remains the same.

**Conclusions:**

The Swedish dental subsidy systems and how dentistry has been treated politically are the results of a chain of events ranging from care for the population's dental health, political doctrines, ‘zeitgeist‚, dental policy, to state finances.

## Introduction

The dental health sector, as part of the Swedish welfare system, originated in 1974.^1,2^ At that time, a dental insurance law was passed, making dentistry free of charge for children and young adults aged 3–19.^3,4^ Since 1974, the dental insurance has undergone three major changes: in 1999, 2002 and 2008, respectively.^5–7^

The major aim of the dental insurance system was to ‘make good dental care financially accessible to all citizens’. Dental care became part of the welfare system, in which the state plays a key role in the protection and promotion of the economic and social distribution of wealth, combined with a responsibility for those unable to provide for themselves. The extent of the state subsidies for care was decided by the dental conditions, and not by the economic situation of the citizen.^1^

The dental insurance of 1974, as well as the following reforms, had a major impact on everything concerning dental care. The insurance made it possible for the majority of Swedes to afford dental treatment of all kinds.^8^ Dentistry also became a part of the political agenda, as an integrated part of the state’s responsibilities and finances. When tracking the development of the dental care systems over time, the influence of party ideology and changes in the general political climate both in Sweden and globally, are detectable.

Lalonde,^9^ who was the Canadian Minister of National Health and Welfare in 1974, proposed a new ‘Health Field’ concept for the understanding of the problems and their causes undermining good health and quality of life for Canadian citizens, and legitimate federal responses ascertained towards these causes. The report is considered to have led to the development and evolution of health promotion, recognising both the need for people to take more responsibility in changing their behaviours to improve their own health, and also the contribution of healthy communities and environments to health. The report was fundamental in identifying health risk behaviours as a determinant of inequalities.

The aims of this paper were to study where in the legislative process the dental politics concerning a national dental insurance and subsidies were formed, and to identify critical impact factors. This is defined as those factors—as described by politicians at various levels, experts and officials, always in the final stages within the framework of a Commission of Inquiry—defining the key issues when identifying the task of a dental insurance system and the way to solve these tasks. Intermediating steps are the necessary funding and legislations necessary for the dental reforms aimed at the defined tasks.

## Materials and methods

The material was collected from the library at the Swedish Riksdag (the parliament) and the Government, and focused on the dental reforms decided by the Riksdag that were preceded by a commission of inquiry. The review search was conducted at the library at Sveriges Riksdag (the Swedish Parliament), which files all public documents related to the political handling of official documents. Library officials at this library were assisting the first author (BF) in finding all of the documents related to the search. BF read all the documents several times in full text. Key documents were then read by the supervisor and second author BA, and thereafter extensively discussed by BF and BA.

The transformation of dental needs, from being a personal matter to that of the society as a whole, started in the early twentieth century. The 1924 parliamentary handling of the dental situation resulted in the first government commission to investigate the conditions and need of dentistry for the whole population. The parliament decided in 1938 that the mouth and singular teeth were part of the larger welfare system and not only a part of medicine, by enforcing a law that slowly should meet the needs of dental care for the entire population.^10^

### Data collection

The usual parliamentary legislative process in the Riksdag was studied:^11^

1. The Government appoints a commission of inquiry and provides guidelines for the questions to be examined and solved.
2. The commission of inquiry submits a proposal (the Swedish Government Official Reports, SOU).
3. The referral of the proposal for consideration.
4. The proposal is presented to the Riksdag, in the form of a Government bill.
5. Proposals from the members of the Riksdag.
6. A parliamentary committee considers the proposals.
7. Debate and decision in the Riksdag.
8. The Government implements the decision.

This strategy identified three major contributors to the process of creating and amending the Swedish national dental insurance system. These include coherent narrative documents that reflect both decisions and underlying reasoning. By tracking the actions from the contributors (1, 2 and 4), it was possible to study where specific ideas and solutions originated.

The documents are also supposed to reflect the input of external ideas, originating from others than the ruling parties (steps 3 and 5).

Each procedural step was examined to identify key issues for all four reforms. Key issues were defined as issues making a decisive impact on the final standpoints. Key issues and which health field each key issue was related to were jointly discussed by the authors. Our principle was to find the main characteristic of a key issue and relate that to a single health field. In a few cases, this was not possible, such as a key issue being general in character and not specific. In those few cases, a key issue could be placed in two health fields.

The following procedure was used for collecting data related to key issues:

1. Reading the material from the archives.
2. Retrieving key issues from the material.
3. Sorting the material, using the modified Health Field Model as a framework.
4. Merging issues and generalisations of concepts.
5. Determine whether the issue exists in all materials.
6. Analysis.

The key issues differed over time, which allowed for tracking changes. For example, the concept ‘dental market’, which is a common concept today, did not exist in 1974.

The analysis of the procedural steps made it possible to identify which contributors that most often provided new ideas and solutions.

### Data analysis.

#### The Health Field Model

The data were analysed and structured using a modified version of the Health Field Model.^11^ In this model, the causative factors for health/illness can be identified within four health fields: Human Biology, Societal-Environment (contextual), Lifestyle (situational) and Health Care Organisation.

Using the Health Field Model to sort the key issues allowed for the analysis of which fields had the most impact on the design of the state dental subsidies.

#### References listed by the commission of inquiry

The role of the commission of inquiry was to transform the political ideas presented by the Government into a working dental healthcare system. Aside from listening to representatives from the dental market, the government agencies, the research community and political parties, the commission also used published information that was available. Another method was only used by the first commission in 1974, when experts wrote documents summing up the state of the art.

The references were sorted into five categories, with subgroups depending on type of data (Table 3):

Odontology

Political science and economy

The Riksdag and the Government

The European Union

Government agencies, including the Swedish Association of Local Authorities and Regions

## Results

Content description of the guidelines for the commissions of inquiry (Guide), the SOU reports from the commissions of inquiry (SOU) and Government bills (Bill) for the dental reforms are presented in Tables 1 and 2. The content was organised in relation to factors within Lalonde’s four health fields.

When a key issue was found in a document it is shown as +. If not found, it is left empty. A horizontal sequence of +, means that the key issues were identified in those documents. This in turn means that the key issue has been regarded important over a number of years and reforms. As an example, the special status of paediatric dentistry (Table 1) has an unbroken chain of + from Guide 1974 to the reform 2008 and is therefore to be considered as an important key issue. Another example is equal competition (Table 2), which first mentioned the guide for the reform 1999 and considered important from there on.

The level of detail was greater in the SOU reports and Government bills than in the guidelines for the commission of inquiry, shown as number of key issues present (+). This was most obvious when it came to the field of Health Care Organisation (Table 2), but also seen in relation to Lifestyle (Table 1).

The reform of 1999 involved a change of system for dental care subsidies, whereas social and political aspirations and general solutions remained the same. Rehabilitation of the mouth and promotion of dental health (Human Biology, Table 1), ability to function, speech and appearance, and dentistry on equal terms (Environment, Table 1), were key issues from 1974 until 2008.

### Key issues within the Health Field Model

#### Human Biology

The views on the fundamental ideas, such as the connection between general and dental health, preventive dentistry, rehabilitation of the mouth, and promotion of dental health, were the same over the years (Table 1). However, the reform of 1999 introduced age-specific selection in the national dental subsidy system.

#### Societal-Environment

The views on the fundamental ideas of ability to function, speech and appearance, dentistry on equal terms and the connection between dental health and socioeconomics have been the same over the years.

#### Lifestyle

Health promotion, i.e., to create a subsidy system that promotes dental health and dental health behaviour, was a recurring theme in the material. The connection between price and dental consumption was stated in the documents from 1999 and onwards.

#### Health Care Organisation

State finances. The financial framework was defined in all reforms from 1999 and onwards (Table 2).

Science. The special status and health results of paediatric dentistry have been cornerstones in Swedish dentistry during all years studied.
Evaluation. A lack of adequate basis for decisions has been a problem in creating a predictable dental subsidy system. Cost, health effectiveness and price became important issues from 1999 and onwards, when dentistry became a free market.
The labour market. In 1974, it was crucial to have enough dentists to meet the demand created by the new subsidy system.
Technical solutions. Social engineering from 1974 and onwards rests on two pillars: preventive dental care and protection against high costs. To control costs and therapies in the high-cost protection system from 1974 to 2008, prior authorisation of dental therapies was used. In 2008, this was replaced with ex-post control. The 1999 reform was the start of selection by age, general illness and disability. In the system run by the state, the subsidy for dental examination for young adults and seniors was determined by the age of the patient. The reform also introduced an alternative capitation system, i.e., Subscription dentistry. Further, the 1999 commission of inquiry introduced a new dental care concept, basic dental care, i.e., all dental care that was not rehabilitating.
The concept of a market. The reform of 1974 rested on the premise that the Public Dental Health Service was competing with private dentists. The solution to this was a planned economy for dental care providers, i.e., price regulation, establishment control and state financial aid to the Public Dental Health Service. However, the patient’s right to choose dentist still remained. In 1999, the report from the commission of inquiry contained proposals constituting a paradigm shift, from a planned economy to a system of market solutions, i.e., equal competition between providers of dentistry, free dental fees, freedom of establishment and price competition. The proposal resulted in the still existing market-like system of dentistry, even though there was a short period of fixed prices for high-cost protected dental care for patients 65 years and older. Controlling for the efficiency was considered important in a free system with state subsidies. In 1999, dental care for patients with certain illnesses, the disabled and some elderly became a part of the general healthcare system. This meant that the responsibilities and costs were transferred from the state to the county councils, a municipalisation of this dental care.

### References listed by the commission of inquiry

Citations used by the commissions of inquiry in 1997, 1998, 2001 and 2007 are listed in Table 3. It shows the references the commissions of inquiry lists in the reports, presented as numbers of documents and year of citations.

#### Odontology

When regarding the importance of epidemiology as equivalent to cited scientific papers, the number of dental documents and their estimated strength did not markedly differ over time.

#### Political science and economy

The 2007 commission of inquiry refers to experience and knowledge outside dentistry in a broader perspective.

#### The Riksdag and the Government

All commissions reuse earlier material and experiences.

#### The European Union

Sweden became a member of the European Union in 1995.

Government agencies, the Swedish Association of Local Authorities and Regions, and all Commissions reused earlier material.

## Discussion

### Methodological considerations

Public health issues, such as dental issues, are often described as general principles (e.g., ‘Good dental health for all’), rather than as actual proposals in political party programs. The work following a Government bill, where a committee considers the proposals with a concomitant debate and decision in the Chamber of the Riksdag, seems to only have a minor impact on what the Government bill will propose.

### Selection of material

The selection of material was made in order to describe how the political system has handled the public funding and organisation of dental healthcare. By using as the selection criterion major reforms primarily related to dental care and dental care systems, the investigated material was narrowed down to decisions taken by the Riksdag, where dentistry was handled separately and not as an item in the state budget or decisions taken by authorities.

By reducing the number of steps in the legislative process (excluding step 3, 5, 6, 7 and 8), there was a risk of losing information from referrals for consideration and proposals from the members of the Riksdag. Our assessment was that the information the other steps would add was negligible and the present information was deemed to be saturated.

At the time of decision on, e.g., the last reform of 2008, there was a wide consensus from the majority of the political parties that the reform should be accepted as it was proposed. One amendment was accepted and that was a stronger writing on collecting and storing dental health data from the clinics. This shows that the political majority system in Swedish Riksdag implies that the governing part does not lay a proposition if it is not more or less guaranteed to pass. There is a wide range of examples of this principle in the past and present political situation in Sweden.

#### The complexity of dental politics

The Swedish dental subsidy systems and how dentistry has been treated politically are the results of a chain of events ranging from care for the population’s dental health, political doctrines, ‘zeitgeist’, dental policy, to state finances. This study of the four dental care reforms does not show what the main idea is, if there is one, regarding the state’s involvement in dentistry. Instead, it is a way to describe the development of dental policy as an evolution under certain basic conditions. The view of the welfare state, providing means to establish function, speech and aesthetically acceptable appearance in the mouth, public finances and the individual’s free choice of dentist, are such conditions.

Another condition is the two equally large parts of the dental market, i.e., the privately and publicly owned dentistry.^8^ General healthcare has been, and still is, dominated by publicly owned healthcare organisations, which especially in the past has created a market where private healthcare organisations have been considered a complementary factor.^12^ The impact created by a large number of patients treated by private dentists puts pressure on politicians to respond to the private dental sector differently from the private general healthcare sector. This means that each new generation of decision makers’ understanding of the dental healthcare organisation and their ideas of solving oral health-related problems will need to adapt to these conditions. This also means that the numbers of possible solutions to the problems are radically reduced. One example of this would be the idea of dental care as part of healthcare. To implement such a system would require radical changes to several basic conditions in dentistry, such as restrictions on the freedom of pricing, health priorities and independent county councils managing the subsidy system with their own interpretation and administrative systems, instead of the state managing the system with the same regulations for the whole country. The dental care that the county council is responsible for today shows the basic differences between the systems.^13^ In political rhetoric, integrating dentistry with the healthcare system is often presented as the ultimate goal. The explanation for not implementing the goal has been a lack of state funding.^14^ This study points out several alternative reasons for dentistry remaining separate from healthcare, such as the complexity of dentistry and dental systems, and the importance of the individual’s right to choose her own dentist.

The reform of 1999 brought many radical changes, and stands as a paradigm shift.^5,15^ It was clear to politicians and dental providers that the subsidy system needed a fundamental change. In brief, the reform of 1999 consisted of municipalisation of the dental care for the elderly, disabled and ill, deregulation of the dental market (free prices, freedom of establishment), introduction of the concept of basic dentistry, more subsidies to young and old adults in the state subsidy system, subscription dentistry, and high-cost protection. This historical evolution of the Swedish dental healthcare system could be described as a good compromise and consensus, rather than confrontation. One example of this would be the inclusion of parts of dental care as part of general healthcare.

The explanation of the structural reform may be the political failure in the recent past. For example, the subscription system was voted down in the Riksdag in 1994.^16^ The need to supplement the results of the commission of inquiry in 1997 with a new commission in 1998 demonstrates the considerable difficulties politicians had in finding a workable solution.^13,15^

Deregulation and high-cost protection suited the private dentists. Municipalisation of some dental care and the political idea of dental care as part of general healthcare were in line with the reasoning of the county councils and patient organisations, such as the Swedish Disability Federation (HSO). Basic dentistry and subscription dentistry suited the Public Dental Health Service.

#### Human Biology, Societal-Environment, Lifestyle and Health Care Organisation

The two fundamental statements made in all examined documents, i.e., ‘dental care on equal terms’ and ‘providing means to establish ability to function, speech and aesthetics’, have set the limits on how to solve dental subsidy systems in Sweden.

Providing means to establish ability to function, speech and aesthetics have had a major impact when designing new dental subsidy systems. Rehabilitation has been considered a basic principle in most healthcare systems.

The reform of 2002 declared basic dentistry to be fundamental for dental health, but while creating a system for people aged 65 or older, it resulted in a system opposite to this. The system was a protection against high costs for citizens 65+, when rehabilitating the mouth using prosthetic dentistry to a maximum cost. On the other hand, basic dentistry was not covered by the 65+ reform.^15^ The idea of basic dentistry, as well as the 65+ system, was abandoned in 2008. This demonstrated a conflict of general ideas and a lack of funding to fulfil political declarations. This is also an interesting example of the saying ‘follow the money’, meaning that funding is the overall ruling principle.

#### Dentistry as a market

Dental care as a market has its historical base.^17^ Prior to 1974, subsidies to organised paediatric dentistry and dental care for conscripts and mothers with infants were the only involvements in the market.^10^ The reform of 1974 led to significant disruption of the dental market by changing the rules in the form of subsidies for patients, establishing limits for private dentists, state financial support to the Public Dental Health Service for the expansion of the business, price regulation of dental tariff, etc.

The various factors and the function of the market have been handled in different ways over the years. But the patient’s right and opportunity to choose dentist have always been fundamental to Swedish dentistry and has never been questioned in the investigated documents.^1^

#### Analysis of citations used by the commissions of inquiry 1997–2007

An interesting finding in the evidence base in the documents reported by the committees of inquiry was the inward perspective on dentistry. The reform of 1974 is a special case and may be seen as a dental system building knowledge about itself.

The lack of trying to use knowledge from other sources than dentistry, or even reporting them, is most remarkable in the 1999 reform. The 90s meant great social and economic changes beyond dentistry: the EU membership and significant changes in the attitude and approach to private providers of education and social services. Documents from that time show a lack of understanding the world outside dentistry.

When analysing the written references to the reform of 1999 (commissions of 1997 and 1998) it becomes clear that the scientific reasons for the changes were weak. Aside from epidemiology describing dental health, there were only two major scientific references: two studies published by the University of Oslo concerning effects of not having dental subsidies for adults. On the other hand, there were eight different memorandums discussing the reform, building on previous works and laws, science, public health and dental care, etc., written by experienced dental professionals.^13,15^

The analysis of references shows that the method used for this study only gives part of the truth when it comes to clarifying motives.^18^ It is rather an indirect description of the ‘zeitgeist’, attitudes and approaches. In order to understand the motives, more in-depth studies using qualitative methods are required.

## Conclusions

It is in general difficult to identify how the discussions in its entirety went regarding the material examined. Dentistry and dental policies do not differ from other political issues when it comes to influencing decision-making processes. Lobbying, networking, personal relationships, etc., influence the political decision-making base, but is seldom reported. The Swedish process in policy making is very strict, transparent and predictable, and guarantees the same and equal handling of different proponents’ views. Taking this in regard, it is in our opinion no reason to assume that the dental policy handling has been processed in a unique way in relation to other political policy makings. Also, this article does not aspire to present the view of policy making outside the political sphere.

However, one must remember that there is a fundamental difference of the ontological nature of natural sciences and human sciences. The kinds of laws and relations that ‘modern’ natural science has established are laws and relations about mathematically formalised entities in models that presuppose causal mechanisms being atomistic and additive in a ‘closed system’. When causal mechanisms operate in real-world social (human) target systems (‘open systems’), they only do it in ever-changing and unstable combinations where the whole is more than a mechanical sum of parts. They can in principle not ever be described in its entire complexity. For a more philosophical framework in this regard, see Marques.^19^

## Figures and Tables

**Table 1:** Content description (present/not present) of the guidelines for the commissions of inquiry (Guide), the Swedish Government Official Reports (SOU) and the Government bills (Bill) for the dental reforms of 1974, 1999, 2002 and 2008

	*1974*			*1999*			*2002*			*2008*		
*Health fields*	*Guide*	*SOU*	*Bill*	*Guide*	*SOU*	*Bill*	*Guide*	*SOU*	*Bill*	*Guide*	*SOU*	*Bill*
*Human Biology*												
Connections dental and general health		+	+	+	+	+	+	+	+	+	+	+
Preventive dentistry		+	+	+	+	+	+	+	+	+	+	+
Rehabilitation of the mouth		+	+	+	+	+	+	+	+	+	+	+
Dental health promotion	+	+	+	+	+	+	+	+	+	+	+	+
Illness, disability and age				+	+	+	+	+	+			
Selection by age				+	+	+	+	+	+		+	+
												
*Societal-Environment*												
Ability to function, speech and appearance	+	+	+	+	+	+	+	+	+	+	+	+
Dentistry on equal terms	+	+	+	+	+	+	+	+	+	+	+	+
Dental health and socioeconomics	+	+		+	+	+	+	+	+	+	+	+
Dentistry in rural area	+								+			
Amalgam				+	+	+		+				
												
*Lifestyle*												
Increased demand	+	+	+					+	+		+	+
Health promotion		+	+	+	+	+		+	+	+	+	+
Personal responsibility for dental health				+							+	+
Price and dental consumption					+	+		+	+		+	+

Abbreviations: +, present; empty, not present.

The content is organised in relation to factors within the health fields of Human Biology, Societal-Environment and Lifestyle.

**Table 2:** Content description (present/not present) of the guidelines for the commissions of inquiry (Guide), the Swedish Government Official Reports (SOU) and the Government bills (Bill) for the dental reforms of 1974, 1999, 2002 and 2008

	*1974*			*1999*			*2002*			*2008*		
	*Guide*	*SOU*	*Bill*	*Guide*	*SOU*	*Bill*	*Guide*	*SOU*	*Bill*	*Guide*	*SOU*	*Bill*
*State finances*												
Financial framework				+	+	+	+	+	+	+	+	+
												
*Science*												
Odontologically motivated/evidence based		+	+				+		+	+	+	+
The special status of paediatric dentistry	+	+	+	+	+	+	+	+	+	+	+	+
Relationship between subsidies and dental health					+	+	+	+				+
												
*Evaluation*												
Inadequate basis for decision					+	+			+	+	+	
Price				+	+	+	+	+	+	+	+	+
Cost effectiveness				+	+	+		+	+		+	+
Health effectiveness				+	+	+		+	+		+	+
												
*Labour market*												
Availability of dentists	+	+	+		+	+	+	+				+
Education directing dentists to the PDHS	+	+	+									
												
*Technical solution*												
Preventive dental care		+	+	+	+	+		+	+	+	+	+
High-cost protection		+	+	+	+	+	+	+	+	+	+	+
Prior authorisation of dental therapies		+	+		+	+		+	+			
Ex-post control of dental therapies					+						+	+
Subsidies for young adults				+	+	+		+	+		+	+
Subsidies for seniors in the state subsidy system				+	+	+	+	+	+	+	+	+
Subsidies for ill, disabled and elderly, for dental care as part of healthcare				+	+	+		+	+	+		
Subscription dentistry (capitation)				+	+	+		+	+	+	+	+
Prioritising basic dental care					+	+		+	+			
												
*Market*												
State financial aid to the PDHS	+	+	+									
Equal competition				+	+	+		+	+	+	+	+
Free dental fees					+	+		+	+	+	+	+
Price regulation		+	+					+	+			
Establishment control	+	+	+									
Freedom of establishment					+	+		+	+		+	+
Control of market efficiency				+	+	+		+	+		+	+
Procurement of services					+	+		+	+			
Municipalisation of certain dental care					+	+		+	+	+		
Patients’ right to choose dentist		+	+	+	+	+		+	+	+	+	+
Price competition					+	+		+	+	+	+	+

Abbreviations: +, present; empty, not present; PDHS, The Public Dental Health Service.

The content is organised in relation to factors within the health field of Health Care Organisation.

**Table 3:** The evidence base used by the commissions of inquiry in 1997, 1998, 2001 and 2007, presented as numbers of articles and the year of citations

	*1997*	*1998*	*2001*	*2007*
*Odontology*				
Published in scientific paper	6	1	2	8
Epidemiology	0	6	8	4
Books, reports, etc.	6	3	4	4
				
*Political science and economy*				
Published in scientific paper	0	0	0	3
Books	0	2	0	2
Reports	0	0	0	4
				
*The Riksdag and the Government*				
Laws	11	11	7	0
Bills, committee reports	7	8	11	5
Commissions of inquiry	4	3	3	3
				
*The European Union*				
Parliament, Council, Court	0	1	0	5
				
*Government agencies, SKL*				
Decisions	1	5	0	4
Statistics	2	8	6	8
Reports	9	0	13	15
Other	0	0	0	1

Abbreviation: SKL, The Swedish Association of Local Authorities and Regions.
